# A Narrative Review of Oral Hygiene and Pulmonary Health Amid Dysphagia: Implications for Feeding Route, Nutrition, and Quality of Life

**DOI:** 10.3390/nu18121888

**Published:** 2026-06-11

**Authors:** Jennifer Hanners Gutierrez, Kenneth Iwuji, Pragya Pandey, Kelly Klein

**Affiliations:** 1 Department of Otolaryngology, Texas Tech University Health Sciences Center, Lubbock, TX 79430, USA; 2 University Medical Center (UMC) Health System, Lubbock, TX 79415, USA; 3 Division of Pulmonary/Critical Care Medicine, Department of Internal Medicine, Texas Tech University Health Sciences Center, Lubbock, TX 79430, USA; kenneth.iwuji@ttuhsc.edu; 4 Department of Conservative Dentistry and Endodontics, King George’s Medical University, Lucknow 226003, Uttar Pradesh, India; w.pragya78@gmail.com; 5 Department of Family and Community Medicine, Texas Tech University Health Sciences Center, Lubbock, TX 79430, USA; kelly.klein@ttuhsc.edu

**Keywords:** nutritional status, quality of life, palliative care, saliva, enteral nutrition, tube feeding, pneumonia, aspiration, deglutition disorders, dysphagia, oral health

## Abstract

Oral health has significant implications for pulmonary outcomes, particularly among individuals with dysphagia who are at risk for aspiration. Moreover, oral health and condition affect nutrition accessibility and status. Inadequate oral hygiene promotes bacterial colonization, plaque accumulation, and aspiration-related respiratory complications. This narrative review aimed to explore current evidence and expert perspectives across palliative medicine, pulmonary and critical care, and dentistry on the role of oral hygiene in supporting pulmonary health and maintaining opportunities for oral nutrition. A comprehensive literature search was conducted through the Texas Tech University Health Sciences Center digital library using Cochrane Library (Wiley), EBSCO Discovery, Embase, Ovid databases, PubMed, SCOPUS, ScienceDirect, Web of Science, and Google Scholar between 14 January 2026 and 1 April 2026. From 1287 identified records, 70 studies were selected to be highlighted in the manuscript after duplicate removal and eligibility screening. Relevant literature was reviewed to examine associations among dysphagia, oral health and condition, oral hygiene and care protocols, feeding route, salivary composition and function, and respiratory outcomes. Emphasis was placed on studies addressing pneumonia, oral versus tube feeding, and evidence-based oral care practices. Findings indicate that pneumonia, depression, and mortality rates are higher in patients receiving tube feeding compared to oral feeding. Evidence-based oral care practices inclusive of mechanical plaque disruption, oral cleansing products (Chlorhexidine, hydrogen peroxide, and sodium bicarbonate), and structured oral hygiene protocols can reduce pulmonary consequences of aspiration and support safer/least risk oral intake. Saliva plays a pivotal role in plaque breakdown, microbial defense, and host immunity; oral feeding helps to preserve salivary function. Results of this review highlight the importance of oral hygiene in both restorative and palliative care contexts. This review establishes a framework for embedding oral cleansing agents and protocols into a nutrition-focused health care infrastructure. Based on the literature analysis and inter- and multidisciplinary clinical expertise of the author group, the manuscript proposes consensus statements intended as expert guidance rather than formal clinical practice guidelines. Adherence to best practices in oral care can mitigate pulmonary consequences of aspiration amid dysphagia, make oral nutrition more accessible and comfortable, sustain opportunities for least risk oral feeding across diagnoses and health care settings, and improve quality of life for patients with dysphagia amid life-limiting illness.

## 1. Introduction

Swallowing is a complex, sensorimotor process that integrates oral, pharyngeal, and esophageal physiology with respiratory coordination and host immune defense [1]. Disruption of this process (i.e., dysphagia) alters a patient’s ability to consume food and liquid by mouth and may increase pulmonary vulnerability [2]. Aspiration, defined as the entry of oropharyngeal or gastric contents into the lower airway, represents one of the most feared complications of dysphagia and can contribute to pneumonia, pneumonitis, respiratory distress, hospitalization, functional decline, and mortality across patients of different ages and with varied diagnoses. Aspiration alone does not inevitably result in pulmonary decline [3]. The biological properties of aspirated material (e.g., microbial load, inflammatory potential, and volume) substantially influence pulmonary outcomes.

The oral cavity functions as a dynamic microbial reservoir. Under conditions of intact salivary flow, effective mechanical clearance, and consistent oral hygiene, the oral microbiome remains relatively balanced and host immunity limits pathogenic overgrowth. In contrast, poor oral hygiene promotes dental plaque maturation, increased bacterial density, and colonization [4]. These organisms may be aspirated during swallowing or reflux-related events. Thus, risk of pulmonary decline is not solely determined by swallow biomechanics, but by the interaction between impaired airway protection, a compromised immune system, and the microbial characteristics of aspirated secretions.

Historically, strategies to mitigate aspiration-related pulmonary harm have emphasized feeding route modification, commonly withdrawal of oral nutrition in favor of tube feeding. This approach is predicated on the belief that bypassing the oropharynx eliminates aspiration and protects the lungs. However, evidence does not support this [5]. Aspiration may occur independent of oral intake through saliva, secretions, or proximally redirected gastric contents. Past studies reveal increased harm from tube versus oral feeding across life-limiting diagnoses [5,6,7]. Tube feeding does not reliably prevent pneumonia and may be associated with persistent or increased respiratory complications and mortality. Findings suggest that aspiration risk is multidimensional and cannot be neutralized by feeding route modification.

Oral hygiene and health have gained increasing recognition as modifiable determinants of pulmonary health, as degraded oral health has been associated with health care complications [8,9]. Unlike disease or injury, oral care practices can be systematically implemented across care settings. Interventions targeting plaque biofilm, salivary preservation, and microbial burden have demonstrated reductions in ventilator-associated pneumonia in critical care populations [10]. Growing evidence indicates that oral hygiene status is an independent predictor of pulmonary decline in both patients who are orally- or tube-fed. Despite this expanding body of literature, oral hygiene remains inconsistently integrated into nutrition-focused dysphagia management. Feeding decisions are frequently made by weighing aspiration risk amid dysphagia without emphasis on oral microbial ecology, secretion management, and salivary function. This separation risks overlooking a biologically plausible and clinically actionable pathway linking oral status to pulmonary outcomes.

The purpose of this narrative review is to underscore evidence from varied disciplines, including dysphagia research, pulmonary medicine, geriatrics, dentistry/endodontics, and palliative care to clarify the oral–pulmonary interface and its implications for nutritional decision-making. Specifically, the authors aimed to: (a) examine the physiological and microbial mechanisms linking oral health to aspiration-related pulmonary decline, (b) highlight the comparative outcomes associated with oral versus tube feeding, and (c) explore the role of structured and collaborative oral hygiene protocols as a modifiable risk reduction strategy across restorative and life-limiting contexts. Consistent with emerging work on interdisciplinary strategies for improving oral health in older adults, these aims position oral hygiene within broader, team-based care in which medical, rehabilitation, nutrition, and dental professionals share responsibility for aspiration prevention and nutritional support in medically complex patients. Rather than a formal systematic review with meta-analysis, authors elected to conduct a structured search and selection process to support a clinically focused discussion of thematic evidence. Authors chose a formal narrative review format given the diversity of populations, interventions, and outcomes in the existing literature. This work proposes a framework for embedding oral hygiene into patient-centered, nutrition-focused care models that prioritize pulmonary protection, feeding opportunity, and quality of life.

## 2. Methods

### 2.1. Review Design

This manuscript was conducted as a narrative review. The review amplifies interdisciplinary evidence from dysphagia research, pulmonary medicine, geriatrics, dentistry/endodontics, and palliative care to examine the oral–pulmonary interface and its implications for nutritional decision-making in patients with dysphagia. Because the objective was to integrate and discuss findings across heterogeneous evidence domains rather than exhaustive effect estimation, a narrative approach was selected. The review was designed and reported with attention to SANRA criteria for the quality of narrative review articles.

### 2.2. Literature Search Strategy

A literature search was conducted through the Texas Tech University Health Sciences Center digital library between 14 January 2026 and 1 April 2026. Databases searched included Cochrane Library (Wiley), EBSCO Discovery, Embase, Ovid databases, PubMed, SCOPUS, ScienceDirect, Web of Science, and Google Scholar. Search concepts included combinations of text words and controlled vocabulary (e.g., MeSH) related to dysphagia, deglutition disorders, aspiration, aspiration pneumonia, oral hygiene, oral health, oral care protocols, bacterial colonization, plaque biofilm, saliva, salivary function, oral versus tube feeding, enteral nutrition, nutritional status, restorative care, palliative care, pulmonary complications, and quality of life. Searches were initially limited to articles published from 2022 onward. When few or no studies were identified for a given topic, the time frame was broadened. Searches drew on indexed scholarly sources; no search of grey literature or unpublished reports was undertaken.

### 2.3. Eligibility Considerations

Priority was given to well-grounded studies published within the previous 10 years, with inclusion of seminal studies when foundational to the topic. Studies were selected if they addressed one or more of the following domains: dysphagia and pulmonary consequences of aspiration; oral health and hygiene in relation to nutrition and nutrition accessibility; oral hygiene and its association with bacterial colonization, plaque accumulation, and aspiration-related pulmonary consequences; oral care protocols; salivary composition and function; interdisciplinary contributions to oral health; oral versus tube feeding; and best practices in restorative and palliative care related to oral care, dysphagia intervention, and preservation of pulmonary health.

These domains were prioritized because they align with the central aim of this narrative review, which is to clarify how dysphagia- and aspiration-related pulmonary risk is shaped not only by swallow physiology and feeding route, but also by oral health status, salivary function, and oral hygiene practices across restorative and palliative care contexts. In general, we deferred studies that lacked clinically relevant information on pulmonary status, respiratory complications, dysphagia and aspiration risk, oral versus tube feeding, or oral health/oral hygiene and its association to nutrition.

### 2.4. Study Selection

**Figure 1:**
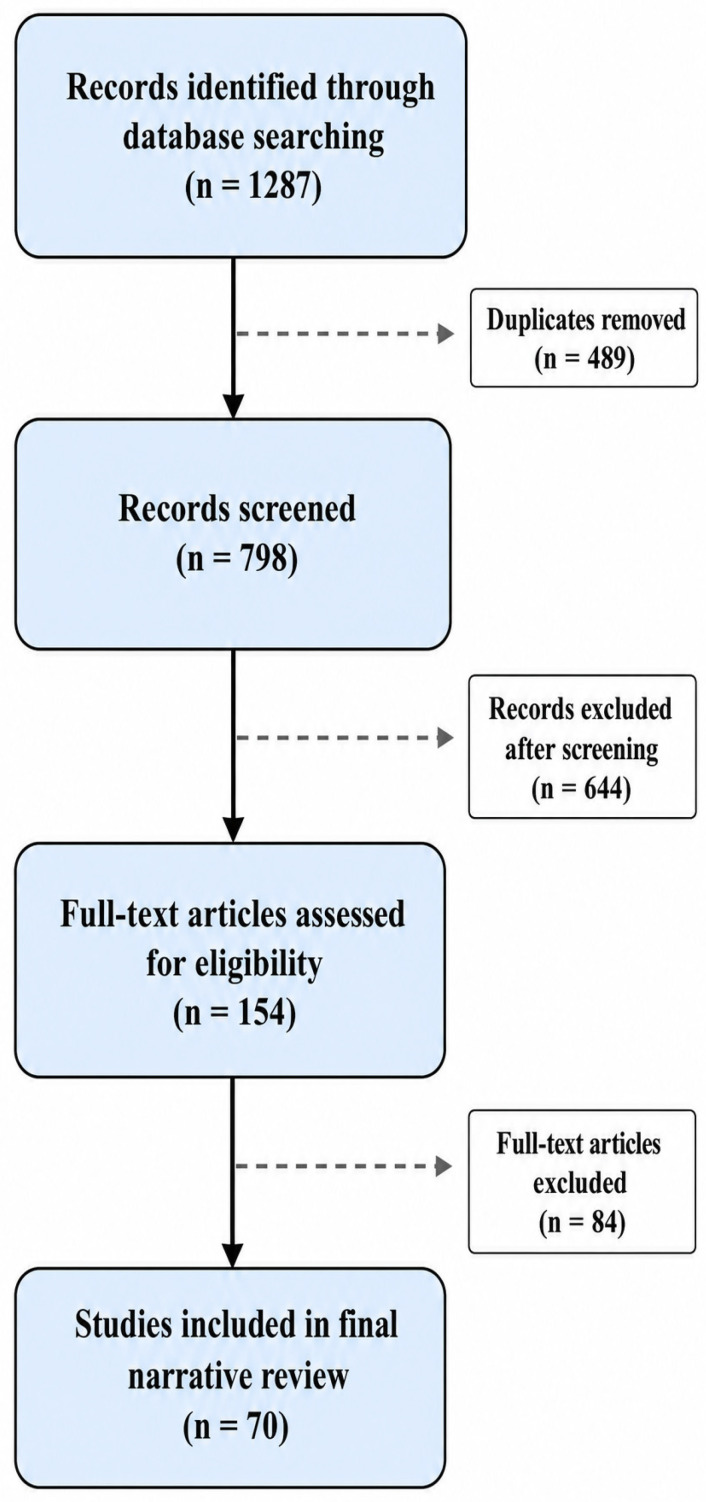
Literature selection flow diagram.

The search identified 1287 records. After removal of 489 duplicates, 798 records were screened. Full-text review was conducted for 154 articles, and 70 studies were included in the final narrative review. The four authors explored findings collaboratively and formulated the consensus statements on the basis of the evidence and inter- and multidisciplinary clinical expertise. Given heterogeneity across populations, interventions, and outcome measures, no formal meta-analysis was undertaken. See Figure 1.

## 3. Results

Given the heterogeneity of the included literature, findings were organized thematically to align with the aims of this narrative review. The following sections synthesize current evidence related to dysphagia physiology and pulmonary vulnerability, oral hygiene and salivary function, feeding route, and interdisciplinary implications for nutritional care.

### 3.1. The Oral–Pulmonary Interface in Dysphagia

***Why is dysphagia a uniquely high-risk condition for pulmonary complications?*** Dysphagia (difficulty swallowing) is a condition that affects many people [2]. Dysphagia can occur across age groups and diagnoses and typically worsens amid polymorbidity at the end of life. One of the feared consequences of dysphagia is aspiration (i.e., foreign material, such as food or liquid, entering the airway and carrying oral pathogens to the lungs). Pneumonia and/or pulmonary decompensation may result from aspiration and, although treatable, impressive pulmonary decline increases risk of mortality.

#### 3.1.1. Phases of Swallowing, Beginning with Oral

Swallowing occurs in phases [11]. The oral phase of swallowing is crucial for bolus (food and liquid) transport from the anterior to the posterior oral cavity. During this phase, food or liquid is propelled to the posterior oral cavity by lingual (tongue) stripping, a progressive force via tongue to palatal contact with the base of tongue helping to push each bolus through the pharynx and into the upper esophagus. Mastication (chewing) is essential for breakdown of solid viscosity. Saliva assists in bolus breakdown and offers oral lubrication which can expedite oral transit. The oral cavity is the conduit to the throat, then the esophagus, then ultimately the stomach. Poor oral hygiene can negatively affect systemic tolerance of nutrition delivered orally [12]. Even in the context of non-oral nutrition, swallowing bacteria-rich saliva can lead to a pulmonary infection. Deficient oral motor capability can result in a patient’s inability to properly breakdown food, yielding risk of airway occlusion of poorly masticated solid viscosity and/or oral residue that breeds harmful bacteria and may contribute to pulmonary compromise over time.

The pharyngeal phase of swallowing is initiated as the bolus transitions from the oral cavity into the pharynx. This phase is characterized by highly coordinated events designed to protect the trachea (airway) while directing the bolus toward the esophagus. Anterior and superior movement of the hyoid bone and elevation of the larynx facilitate epiglottic inversion and closure of the laryngeal vestibule. These layered airway-protective mechanisms are essential for preventing entry of material into the airway. Pharyngeal constriction prompts a sequential stripping wave that clears the bolus through the pharynx, toward the esophageal sphincter which must relax to allow each bolus into the upper esophagus. Breakdown in the pharyngeal phase may occur when swallow initiation is delayed, pharyngeal constriction is suboptimal, and/or airway closure is incomplete. Food or liquid may pool in the valleculae or pyriform sinuses which can contribute to aspiration of pharyngeal residue. Of note: aspirated material during this phase is not limited to food or liquid. Saliva and oral secretions may also be aspirated and, amid poor oral hygiene and colonized pathogenic bacteria, pulmonary infection may ensue. This risk is amplified in patients with reduced laryngeal sensation, impaired cough response, or diminished respiratory reserve.

The esophageal phase of swallowing begins once the bolus passes through the upper esophageal sphincter and enters the esophageal body. Bolus transport during this phase is driven by coordinated peristaltic contractions that push material to the stomach. While this phase is anatomically distal to the airway, esophageal dysfunction can still contribute to pulmonary decline. Impaired esophageal clearance, dysmotility, or lower esophageal sphincter dysfunction may result in retrograde movement of contents into the pharynx, particularly when the patient is supine or has reduced esophageal tone. Regurgitated material may then be aspirated, often outside the context of an active swallow.

When esophageal clearance is inefficient or abnormal, repeated exposure of the pharynx to proximally redirected contents can exacerbate inflammation, alter sensation, and disrupt the timing of subsequent swallows. Retrograde flow of esophageal contents may further impair airway protection via increasing the risk of aspiration of upper gastrointestinal contents and bacteria-laden oropharyngeal secretions. In patients with dysphagia, especially those receiving tube feeding, esophageal phase and upper gastrointestinal impairment underscores why aspiration risk is not eliminated by bypassing oral intake alone [13].

Dysfunction with any phase of swallowing can elevate pulmonary risk. Poor oral hygiene is a contributing factor to pulmonary ingestion of pathogens. The interaction between impaired swallow physiology and oral health status defines a critical oral–pulmonary interface. Disruption at one phase may compound vulnerability at another, reinforcing the importance of viewing dysphagia not as a single mechanical failure but as a condition shaped by oral hygiene, salivary function, neuromuscular coordination, and feeding practices.

#### 3.1.2. Diagnoses Associated with Dysphagia

Dysphagia does not occur as an isolated disorder but rather emerges across a wide range of medical, neurological, developmental, and structural conditions. Because swallowing relies on optimal neuromuscular coordination, sensory input, respiratory stability, and structural integrity, disruption in any of these domains may compromise functional oral intake. The presence of dysphagia often reflects the broader physiological burden of disease, injury, or developmental immaturity—rather than a single focal impairment. Its presence frequently co-occurs with increased pulmonary vulnerability and altered nutritional tolerance.

Neurological diagnoses represent one of the most common etiologic categories associated with dysphagia. Stroke [14], traumatic brain injury [15], and neurodegenerative diseases disrupt cortical and brainstem networks responsible for the timing and coordination of swallowing. This disruption may result in delayed pharyngeal engagement in swallowing/swallow initiation, reduced airway protection, and impaired bolus clearance. Progressive disorders such as Parkinson’s disease, Amyotrophic Lateral Sclerosis (ALS), and multiple sclerosis are associated with worsening dysphagia over time due to decline in motor control, sensory feedback, and cough strength [16]. Dysphagia in these populations can contribute to pneumonia, malnutrition, and reduced quality of life.

Dysphagia is also prevalent in individuals with respiratory disease and critical illness [17]. Tracheostomized patients and patients requiring ventilator support to achieve optimal vital capacity may experience deficient hyolaryngeal excursion, poor swallow–respiratory coordination, and diminished laryngeal sensation. Respiratory conditions such as chronic obstructive pulmonary disease (COPD) may compromise swallow safety by limiting respiratory reserve and increasing the likelihood of food/liquid inhalation amid breathing and deglutition [18]. Silent aspiration (i.e., airway invasion without overt/clinical indicators of dysphagia) is prevalent amid respiratory-related health care complications.

Structural and oncologic conditions of the head and neck represent another primary category of diagnoses that may result in dysphagia as a complicating factor [18]. Tumors of the oral cavity, pharynx, or larynx and medical management of oropharyngeal cancer (e.g., surgical resection, radiation therapy, chemotherapy), may alter anatomy, reduce tissue compliance, and impair salivary production. Bolus formation, propulsion, and clearance are often negatively impacted and pharyngeal residue may pose risk of aspiration. Reduced salivary flow and degraded mucosal integrity further promote bacterial colonization, reinforcing the relevance of oral health in mitigating pulmonary complications in this population.

Systemic and gastrointestinal diseases may also contribute to dysphagia. Esophageal dysmotility, gastroesophageal reflux, and conditions such as liver disease, renal disease, and sepsis can impair neuromuscular performance, cognition, and endurance and can lead to deficits in swallow functionality [19,20,21]. Retrograde movement of esophageal or gastric contents into the pharynx increases the risk of aspiration, particularly in patients with reduced airway sensation or diminished protective reflexes. These mechanisms highlight how dysphagia-related aspiration risk may exist even when oral intake is restricted or limited.

In pediatric populations, dysphagia frequently reflects developmental immaturity or congenital conditions rather than acquired disease. Prematurity is a leading risk factor, as infants born preterm often lack suck-swallow-breathe coordination, adequate oral motor strength, and feeding endurance [22]. Dysphagia also commonly occurs amid cerebral palsy [23], genetic syndromes [24], cleft palate and craniofacial anomalies [25], and congenital heart disease [26]. Feeding difficulties in these populations may persist across development and are often accompanied by gastroesophageal reflux, chronic lung disease, or prolonged reliance on tube feeding. Limited oral feeding experiences during critical development periods may further influence oral sensory processing and skill acquisition—with downstream implications for swallow functionality and nutrition.

Across the lifespan, dysphagia should therefore be conceptualized as a condition shaped by diagnosis, disease trajectory, and cumulative physiological stressors. When arising from prematurity, neurological injury, critical illness, or progressive disease, dysphagia alters how individuals manage food, fluids, and their own secretions. The interaction between underlying diagnoses, oral health status, and pulmonary vulnerability reveals the clinical significance of dysphagia and reinforces the need for integrative, interdisciplinary approaches to assessment and management.

### 3.2. Oral Hygiene as a Modifiable Risk Reduction Strategy

***How does oral hygiene influence aspiration-related pulmonary outcomes?*** Oral hygiene is increasingly recognized as a modifiable determinant of pulmonary health and is especially important in populations vulnerable to dysphagia and deficient airway protection. The oral cavity serves as a major ecological haven for microorganisms, many of which are routinely transported to the pharynx and lower airway through swallowing, aspiration, or reflux-related events. When oral hygiene is inadequate, this microbial reservoir shifts toward higher bacterial density, increased plaque biofilm burden, and greater representation of potentially pathogenic organisms. These changes alter the biological characteristics of material that may enter the respiratory tract and influence pulmonary outcomes. In contrast to fixed neurological or structural contributors to dysphagia, oral hygiene status is amenable to intervention across the continuum of care—including tertiary care, long-term care, home health, and palliative care settings. Importantly, oral hygiene exerts its effects upstream of pulmonary infection, modifying the microbial content of secretions that may be aspirated rather than directly altering swallow physiology. This distinction is critical. Oral care does not prevent aspiration, but it does reduce the pathogenicity of aspirated material. Oral hygiene represents a biologically plausible and clinically feasible target for reducing pneumonia risk in medically vulnerable individuals [27].

#### 3.2.1. Saliva: Types, Biological Functions and Clinical Relevance

Saliva is a multifunctional, biological fluid that plays a vital role in oral homeostasis, swallowing efficiency, and immune defense. It is produced by the paired major salivary glands (parotid, submandibular, and sublingual) and numerous minor glands distributed throughout the oral mucosa [28]. These glands secrete saliva with distinct compositional profiles that collectively support oral and systemic health.

Parotid gland secretions are predominantly serous and contribute high concentrations of digestive enzymes such as α-amylase, along with antimicrobial proteins such as lysozyme, lactoferrin, histatins, defensins, and salivary peroxidases. These components inhibit bacterial growth, disrupt microbial membranes, and limit colonization by pathogenic species. In contrast, the submandibular and sublingual glands produce mixed serous-mucus secretions enriched with mucins, primarily MUC5B and MUC7. These glycoproteins confer viscosity and elasticity, facilitating bolus cohesion, lubrication, and epithelial protection [29,30]. Minor salivary glands provide continuous baseline secretions that maintain mucosal hydration and local immune surveillance.

Beyond facilitating mastication and bolus transport, saliva performs several functions relevant to pulmonary risk. It buffers oral pH, dilutes microbial concentrations, promotes mechanical clearance of bacteria, and limits adherence of microorganisms to oral surfaces. Salivary flow also modulates the structure and composition of oral biofilms. When salivary quantity or quality is reduced (e.g., due to aging, polypharmacy, dehydration, radiation therapy, systemic illness, reduced oral intake), these protective mechanisms are compromised. Hyposalivation is associated with increased bacterial density, accelerated plaque accumulation, altered oral microbiome composition, and increased prevalence of opportunistic pathogens [31].

In individuals with dysphagia, the clinical relevance of saliva is magnified. Saliva is swallowed frequently throughout the day, independent of eating or drinking. When oral hygiene is poor and salivary flow is reduced, saliva may serve as a concentrated vehicle for pathogenic microorganisms. Aspiration of saliva alone, without food or liquid, has been identified as a contributor to pulmonary infection, especially amid deficient airway sensation, diminished cough, altered swallow–respiratory coordination, and overall suboptimal swallow biomechanics. Data on salivary makeup and biological use explain why oral hygiene and salivary preservation remain relevant even in patients who are not receiving oral nutrition. Consistent oral hygiene and, when possible, oral intake help preserve salivary function and oral sensory stimulation, supporting bolus formation, secretion clearance, and tolerance of oral feeding.

#### 3.2.2. Bacterial Colonization, Oral Pathogens, and Plaque Biofilm, and Oral Cleaning Agents

Bacterial colonization of the oral cavity is a continuous biological process shaped by host factors (salivary flow and composition, mucosal integrity, immune function), behavioral factors (oral hygiene frequency and technique), and clinical context (hospitalization, mechanical ventilation, dependence for cares). Colonization becomes clinically consequential when microbial density increases and biofilm maturation occurs on teeth, dentures, tongue dorsum, and oral mucosa. Dental plaque is a structured polymicrobial biofilm embedded in an extracellular matrix, with resistance to shear forces and reduced susceptibility to antimicrobial agents.

This oral–pulmonary reservoir concept is supported by evidence that poor oral hygiene and impaired oral clearance can shift the oral ecosystem toward enrichment with potential respiratory pathogens [32,33]. In older adults in residential care, microbiologic studies and systematic reviews have identified organisms commonly associated with respiratory infection (e.g., Staphylococcus aureus (including MRSA), Enterobacteriaceae, Pseudomonas aeruginosa, and Candida species) in oral specimens of patients who develop aspiration pneumonia. Data reveal lower aspiration pneumonia incidence in cohorts receiving professional oral care compared with usual care [34,35,36]. In parallel, molecular studies in hospitalized and critically ill populations support microbial concordance between pathogens recovered from lower-airway specimens (e.g., bronchoalveolar lavage) and organisms isolated from dental plaque, consistent with aspiration of colonized secretions or microaspiration as a plausible pathway [37,38]. Importantly, a single universal microbial threshold for aspiration pneumonia is not established; rather, risk is best conceptualized as an interaction between aspiration events and colonization burden in the context of impaired host defenses [39,40].

Oral disease burden may further amplify this reservoir, as periodontal inflammation increases biofilm retention and inflammatory mediators, and poor oral health has been associated with respiratory diseases [41,42]. Beyond periodontal inflammation, untreated caries and residual roots can increase biofilm-retentive surfaces and contribute to chronic oral discomfort, which may reduce effective mastication and compromise dietary intake quality. While direct causal links between caries-specific pathogens and aspiration pneumonia are not established, caries burden can function as a marker of oral disease severity and hygiene limitation in medically complex patients [36,43,44]. Collectively, these findings provide a mechanistic rationale for structured oral hygiene as a scalable intervention to reduce pathogen load and improve mucosal integrity, thereby mitigating aspiration-associated infectious risk amid dysphagia.

Studies that have investigated the impact of antiseptic or oral cleansing agents have mostly focused on mechanically ventilated intensive care unit (ICU) populations. Past research has analyzed the association of oral care to ventilator-associated pneumonia (VAP). Well-grounded clinical trials tested the effects of reduction in oral microbial burden on pulmonary infection risk. Across this literature, a key distinction is that chemical agents primarily act by reducing microbial load and altering biofilm structure. Chemical agents via swab or swish can be a key adjunct to mechanical plaque disruption (brushing).

Chlorhexidine (CHX) has broad antimicrobial activity and substantive binding to oral tissues, supporting sustained effects. Multiple meta-analyses and reviews have reported reductions in VAP incidence with CHX products and protocols when used within structured oral care regimens. For example, a 2021 meta-analysis (7 studies, 1424 participants) reported a statistically significant (*p* = 0.005) reduction in VAP with CHX oral care combined with brushing [45]. A 2024 comparative analysis of oral hygiene methods concluded that tooth brushing combined with 0.12% CHX ranked highest for reducing VAP incidence, followed by 0.12% CHX alone and brushing alone [46]. Other recent syntheses, including a 2024 meta-analysis [47], have found no significant advantage of CHX for preventing VAP and raised concerns about potential harms, illustrating ongoing uncertainty. Research suggests that in some ICU and non-ICU populations, patients who received CHX appeared more likely to die than those who did not, but this observation comes mainly from secondary or post hoc analyses and was not the primary question those studies were designed to answer. Mortality concerns therefore rely on indirect signals from heterogeneous cohorts, and no clear biological mechanism has been demonstrated to explain how standard intraoral CHX concentrations would directly increase the risk of death. Moreover, mortality in critically ill patients is driven by multiple competing factors, so it is difficult to separate the specific contribution of CHX exposure from the underlying severity of illness, comorbidities, and other aspects of care.

Hydrogen peroxide (H_2_O_2_) has been evaluated less extensively than CHX but has a clearer mechanistic rationale as an oxidizing agent that can disrupt bacterial membranes, reduce anaerobic organisms, and facilitate loosening of debris. A 2016 randomized trial reported that hydrogen peroxide mouthwash reduced VAP compared with normal saline in mechanically ventilated patients [48]. Temporary use of hydrogen peroxide (3%) was also supported by a 2024 review [49], and concentrations of 1–1.5% have been shown to be effective in reduction in oral bacterial load [50]. More recent research continues to explore hydrogen peroxide-based oral care as an alternative strategy in ICU settings, including studies designed to compare hydrogen peroxide regimens with CHX-based (0.01–0.2%) standard care and to evaluate effects on lower airway microbial colonization [51]. While these efforts reflect ongoing clinical interest, the current clinical evidence remains narrower than for CHX, with fewer large multi-center trials and meta-analytic syntheses. Therefore, hydrogen peroxide can be described as a plausible antimicrobial adjunct with supportive trial data in ventilated patients, but broader conclusions about pneumonia prevention across settings should be made with discernment until additional data is made available.

Sodium bicarbonate (NaHCO_3_) solutions are used clinically to alkalinize the oral environment, thin and mobilize tenacious secretions, and facilitate mechanical removal of debris—properties that may be relevant in xerostomia, mucosal coating, or thick oral secretions. Its antimicrobial activity is not typically framed as broad-spectrum bactericidal potency comparable to CHX; rather, sodium bicarbonate may influence microbial ecology through pH modulation and improved clearance of secretions and biofilm material. Evidence evaluating sodium bicarbonate in relation to pneumonia outcomes is comparatively limited and often embedded within multi-component protocols. For example, a 2022 study examining oral cavity alkalinization using sodium bicarbonate oral rinse in combination with CHX reported a lower incidence of VAP compared with CHX alone [52]. In neonatal critical care, a 2021 study evaluated combined oral care using colostrum and sodium bicarbonate for prevention of neonatal VAP, illustrating use of NaHCO_3_ within bundled neonatal oral care strategies rather than as an isolated intervention [53]. Overall, NaHCO_3_ appears best characterized as an agent that may improve oral clearance conditions and support mechanical cleansing. The current evidence is insufficient to claim consistent reductions in pneumonia incidence when sodium bicarbonate is used alone. See Table 1 for a summary of oral cleansing agents and protocols.

When available, typical concentrations, frequencies of administration, and protocol durations for oral cleansing agents are summarized in Table 2.

### 3.3. Feeding Route Does Not Eliminate Aspiration Risk

Feeding route is frequently used as a strategy for reducing pulmonary complications in individuals with dysphagia. In clinical practice, the transition from oral feeding to tube feeding is often justified by the belief that bypassing the oropharynx reduces aspiration risk and protects the lungs. However, aspiration does not solely occur via ingestion of food or liquid amid oral feeding. Impaired airway protection, diminished cough effectiveness, suboptimal secretion management, upper gastrointestinal abnormalities such as reflux, and overall disease burden remain unchanged by feeding route modification. Tube feeding does not eliminate pulmonary risk and may, in certain populations, be associated with worse outcomes [54]. A growing body of literature has reported either no benefit or increased harm associated with tube feeding when compared with oral feeding, including careful hand oral feeding. Findings warrant reconsideration of selection of tube feeding as a singular pulmonary-protection strategy.

#### 3.3.1. Oral Feeding Versus Tube Feeding: Comparative Clinical Outcomes

In advanced dementia, multiple cohort studies and systematic reviews have failed to demonstrate a reduction in aspiration pneumonia or improved survival with tube feeding. Cintra et al. (2014) reported increased pneumonia and mortality in patients with advanced dementia receiving tube feeding versus oral feeding [55]. Similarly, a 2021 systematic review and meta-analysis investigated tube feeding use in the context of advanced dementia and found no benefit to longevity [6]. Moreover, increased health care complications were noted in patients who received tube feeding.

Additional studies further amplify this concern. In a retrospective cohort of 764 patients with advanced dementia (2015–2019), nasogastric (NG) feeding was compared with careful hand oral feeding [56]. Survival did not differ significantly between groups at 1 year. However, pneumonia occurred more frequently in patients receiving NG nutrition (60% vs. 48%; *p* = 0.004), and NG nutrition remained independently associated with pneumonia risk after adjustment (adjusted HR 1.41; 95% CI 1.08–1.85). In a large prospective audit of patients who underwent gastrostomy tube (G-tube) placement, primarily percutaneous endoscopic gastrostomy (PEG) placement—mortality was reported at 8% at 30 days, 16% at 3 months, and 35% at 12 months [57]. Early deaths were attributed to respiratory causes. Data underscore that tube feeding via gastrostomy does not eliminate pulmonary risk and support greater discernment of patient selection and clearer goals-of-care discussions prior to gastrostomy placement. Prospective data across life-limiting diagnoses further extend these findings. In a cohort of 65 patients with dysphagia and terminal illness, pneumonia occurred in 79% of tube-fed participants versus 12% of participants who received oral nutrition. After adjustment for age and mortality risk, tube feeding remained strongly associated with pneumonia (adjusted OR 19.28; 95% CI 4.5–109.6; *p* < 0.01) [5].

Across diagnostic categories and study designs, several consistent observations emerge: (a) tube feeding does not reduce pneumonia incidence amid advanced dementia; (b) survival benefit from tube feeding in advanced or terminal illness has not been consistently demonstrated; (c) pneumonia may occur more frequently in patients who are tube-fed versus oral-fed, and (d) early mortality following placement of tube feeding is common in medically fragile populations and is frequently respiratory in origin. Default use of tube feeding versus oral feeding to prevent aspiration is not an evidence-based strategy and is not a dependable pulmonary-protective strategy in advanced illness. Nutritional decision-making requires consideration of underlying disease trajectory, goals of care, and the multidimensional nature of aspiration risk.

#### 3.3.2. Persistent Pulmonary Risk, Mortality, and Psychosocial Outcomes Despite Tube Feeding

Pulmonary risk persists following initiation of tube feeding because contributors to aspiration extend beyond ingestion or oral pathogens carried by food or liquid to the lungs. Patients continue to swallow saliva regardless of the feeding route, and deficits such as impaired laryngeal closure and diminished cough remain unchanged. Moreover, tube feeding introduces potential for reflux-associated aspiration. Nasogastric (NG) tubes may compromise lower esophageal sphincter function, and delayed gastric emptying (common in critical illness, neurological injury or disease, and advanced age) may increase the likelihood of retrograde flow. Gastrostomy tube (G-tube) feeding does not eliminate this risk when esophageal dysmotility or reduced sphincter tone is present. Thus, tube feeding may alter the substrate aspirated but does not eliminate aspiration risk.

Large-scale research studies report pulmonary and mortality risk after tube placement. In a retrospective cohort analysis from England (2007–2019), 87,682 patients underwent PEG placement [58]. Aspiration pneumonia occurred within 7 days of PEG placement in 8–10% of participants across study intervals, and 30-day mortality ranged from approximately 5–13% over time. The reported decline in early mortality and aspiration pneumonia that occurred in later versus early study years was attributed to evolving selection practices (e.g., fewer PEG placements amid advanced dementia and delayed placement following acute stroke) rather than to any intrinsic protective effect of tube feeding. Even in contemporary practice with improved selection, clinically significant early aspiration pneumonia and mortality remain documented outcomes, further revealing that PEG placement does not neutralize pulmonary vulnerability. A 2023 systematic review focused on predictors of early mortality after PEG placement and reported that 30-day mortality ranged from 2.4% to 23.5%, with older age and dementia most frequently associated with early mortality [59].

Oral hygiene is clinically relevant within this discussion because tube feeding does not make the oral cavity physiologically irrelevant. Tube-fed patients continue to generate and swallow secretions, and oral status can deteriorate when oral intake ceases or when oral care is deprioritized. Recent evidence indicates that oral hygiene can independently stratify pneumonia risk even among participants who are tube-fed. A 2025 study investigated hospitalized adults who underwent oropharyngeal assessments via endoscopy [60]. Tube feeding was independently associated with pneumonia (adjusted OR 2.50; 95% CI: 1.36–4.60; *p* = 0.003), and poor oral hygiene was independently associated with pneumonia (adjusted OR 3.48; 95% CI: 1.84–6.56; *p* < 0.001). The authors reported that poor oral hygiene exacerbated pneumonia risk within feeding subgroups, including participants who were fed via NG-tube or PEG, supporting the assertion that oral hygiene markedly modifies pneumonia risk—even when nutrition is delivered by tube. Supportive data in long-term care also reveal that oral care interventions can reduce pneumonia burden among patients who are tube-fed. One unique research study investigated the impact of an oral care protocol on bedridden, older adults who received tube feeding [61]. The oral care protocol was associated with a reduction in pneumonia incidence from 1.20 to 0.45 over the observation period. This study points to oral care being a modifiable determinant of pulmonary outcomes in patients who receive nutrition via tube feeding.

Physiology and translational research converge on this conclusion: Tube feeding changes the route of nutrient delivery but does not eliminate pneumonia risk, because aspiration pathways persist (e.g., secretions, reflux, retrograde flow) and underlying airway defense impairments remain. Past studies demonstrate early aspiration pneumonia and mortality amid tube feeding. Emerging evidence indicates that oral hygiene status significantly impacts pneumonia risk, reinforcing that pulmonary protection may not be achieved through feeding route modification.

#### 3.3.3. Swallow Deconditioning and Oral Neglect in Patients Who Are Tube-Fed

Tube feeding modifies nutrient delivery but does not suspend aerodigestive physiology. Saliva production, spontaneous swallowing, and airway oropharyngeal secretions continue regardless of feeding route. When oral intake is withdrawn, however, the functional demands placed on the swallowing mechanism change substantially. The act of eating provided repetitive, task-specific activation of swallow musculature and reinforces neuromuscular coordination between respiration and deglutition. Removal of oral feeding reduces this repetitive sensorimotor activity and may contribute to progressive swallow deconditioning in individuals already vulnerable due to neurological injury or disease, respiratory compromise, polymorbidity, or prolonged hospitalization for alternative reasons.

Reduced swallowing frequency (that occurs with consistent stimulation in the context of oral feeding) has physiological implications. Spontaneous and functional/volitional swallows help to clear secretions. When swallow frequency declines or swallow biomechanics lack the ignition offered by eating by mouth, pooling of secretions in the oropharynx may occur which can increase the likelihood of aspiration [62]. In this context, tube feeding does not eliminate aspiration, but rather, it alters the dynamics of secretion management. Tube feeding may also shift clinical priorities in ways that inadvertently affect oral status. Once nutrition is delivered enterally, attention often centers on caloric adequacy, tube patency, and gastrointestinal tolerance. The oral cavity may receive less structured surveillance when it is no longer the route of feeding. In addition, absence of mastication and gustatory stimulation can reduce salivary flow and alter oral comfort, particularly in the presence of polypharmacy or systemic illness.

**Figure 2:**
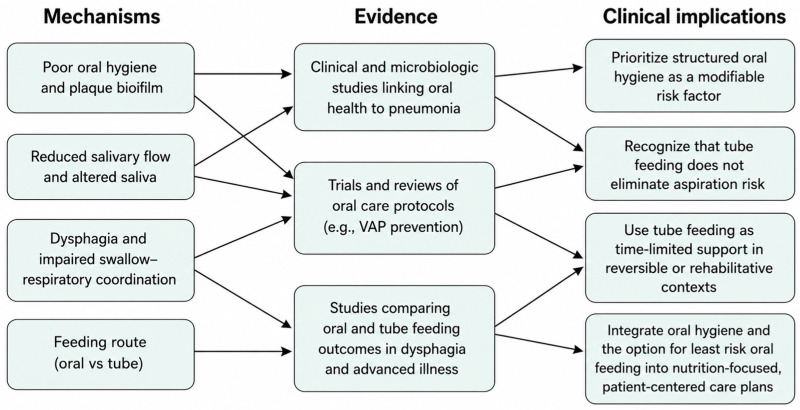
A conceptual framework linking oral hygiene, saliva, dysphagia, and feeding route to aspiration-related pulmonary outcomes and nutrition decisions.

Oral feeding typically requires upright positioning, structured mealtimes, and caregiver engagement. The transition to exclusive tube feeding may reduce these structured periods of activity, potentially contributing to reduced mobility and generalized deconditioning. Limited mobility [63] and frailty are associated with pulmonary vulnerability, suggesting that feeding route decisions may indirectly influence respiratory outcomes through effects on overall functional status. Swallow deconditioning, altered secretion clearance, reduced oral surveillance, and functional decline illustrate that tube feeding does not disengage the upper aerodigestive tract from risk. Instead, feeding route modification may alter the pattern of exposure while leaving core vulnerabilities intact. See Figure 2.

### 3.4. Implications for Nutrition-Focused, Patient-Centered Care

Evidence reveals that feeding route modification alone does not eliminate pulmonary consequences that may result from aspiration, and oral status is clinically relevant amid oral or tube feeding. Nutritional decision-making amid dysphagia should extend beyond caloric adequacy and feeding route selection to include structured evaluation of the patient’s oral health, patient goals, and anticipated trajectory of disease or injury. A nutrition-focused, patient-centered approach integrates oral care, feeding strategy, and quality-of-life considerations into a unified care plan rather than treating them as independent domains. A multidisciplinary, collaborative approach between diverse specialists such as speech therapists, neurologists, nutritionists, pulmonologists, nurses, and rehabilitation professionals can elevate oral hygiene and aspiration prevention in dysphagic patients across different clinical settings.

Dysphagia intervention is often prioritized for pulmonary risk reduction, yet its clinical success is ultimately judged by nutrition and hydration adequacy—whether patients meet energy/protein needs, maintain fluid balance, and sustain an acceptable diet/food variety and eating experience. Nutrition guidelines for neurological disease recognize dysphagia as a major driver of malnutrition and dehydration risk and support routine nutrition-risk screening with timely dietetic input to individualize texture/fluid modification and feeding strategies [64]. In this context, oral health is best considered a practical, modifiable determinant of “nutrition feasibility.” Systematic reviews in older adults show consistent associations between poorer oral health and malnutrition risk as assessed by standard tools (e.g., Mini Nutritional Assessment [MNA], Subjective Global Assessment [SGA]), reflecting contributions of impaired dentition/oral function and symptom burden to reduced intake [43,44]. Emerging evidence further links oral health indicators with oropharyngeal dysphagia, suggesting that oral status may influence rehabilitation trajectories and tolerance of prescribed textures rather than operating only as a parallel comorbidity [65]. Accordingly, oral assessment and structured oral care should be viewed as enabling components of nutrition therapy in the context of dysphagia, with potential to improve dietary adequacy, hydration, and patient-reported eating experience by addressing modifiable oral barriers.

#### 3.4.1. Nutritional Risk Screening and Structured Oral Care Protocols

Nutrition risk screening in medically complex patients typically incorporates weight change, intake adequacy, functional status, and disease burden. Oral health and condition should be incorporated into the assessment of any patient with dysphagia because it influences tolerance of oral intake, secretion management, and overall comfort. Documentation of dentition status, mucosal integrity, salivary adequacy, presence of debris or coating, and patient ability to participate in oral hygiene provides tangible and clinically useful information for both dietetic and dysphagia management. Moreover, assessment of oral health and provision of oral cleansing is not solely the responsibility of nursing but a shared responsibility of health care providers.

Oral care protocols in hospitalized and long-term care settings commonly consist of: (a) mechanical plaque disruption twice daily/*every 12 h* (brushing) and (b) implementation of oral cleansing *every 2–4 h* (at the very least, every 12 h [51]) with an evidence-based cleansing agent [52]. Tooth brushing with a soft-bristled toothbrush morning and evening is standard practice. Brushing should include the gingival margin and tongue dorsum. In addition, oral cavity cleansing with an effective agent (e.g., diluted hydrogen peroxide/hydrogen peroxide [above 1% and recommended 1–1.5%] rinse or CHX [0.01–0.2%]) after meals supports clearance of food residue, improves oral comfort, and cleanses the mouth in preparation for the next meal—reducing likelihood of pulmonary ingestion of oral pathogens. Mouth moisturization is also an essential part of oral care and health. For denture wearers, removal and cleaning of dentures daily, with overnight storage outside of the mouth, is recommended. Incorporating oral care into the nutrition assessment reframes it from an adjunct hygiene task to a determinant of feeding tolerance, comfort, and pulmonary vulnerability. See Table 2 for a summary of evidence-based oral protocols.

#### 3.4.2. Dental Involvement in Critical-Care Settings

In critical-care settings, dental involvement is typically oriented toward structured bedside assessment, triage, and source control rather than definitive restorative or endodontic treatment [66,67]. Hospital dentistry reviews emphasize that nurse-managed oral assessment tools may fail to detect caries and periodontal disease. Past research reports medically complex patients have increased incidence of oral pathology that contributes to pain, mucosal trauma, bleeding, and biofilm retention [66]. A dentist-led evaluation can identify urgent problems (e.g., advanced caries with pain, suspected odontogenic infection, periodontal abscess, severe mucosal injury from sharp tooth edges, heavily colonized dentures, or highly mobile teeth posing airway/aspiration hazard) [66,68]. Dental providers can guide feasible interventions such as smoothing sharp edges, placing temporary protective restorations when appropriate, optimizing denture management, and co-developing implementable oral care protocols with nursing staff (e.g., suction-assisted mechanical cleaning and xerostomia management) [66,67]. More invasive procedures (e.g., comprehensive periodontal therapy, elective extractions, endodontic treatment, prosthetic rehabilitation) are commonly deferred until clinical stability permits. However, early recognition and mitigation of urgent oral disease sources may improve comfort and support nutrition-based goals while potentially reducing the pathogenicity of aspirated secretions.

#### 3.4.3. Supporting Oral Feeding or Least Risk Oral Feeding When Feasible

In many cases, based on research, oral feeding offers a safer context for nutritional delivery even in the context of dysphagia. Oral feeding can also be rehabilitative. Oral feeding should be heavily considered when at all possible versus default use of tube feeding if a patient has demonstrated signs of dysphagia clinically or during an instrumental examination of swallowing. Oral feeding or careful hand feeding with individualized texture modification, compensatory strategies, and dysphagia intervention by an expert such as a speech–language pathologist are associated with lower incidence of pneumonia as compared to tube feeding amid critical or terminal illness. Multidisciplinary protocols in progressive neurological conditions with prominent bulbar involvement, such as amyotrophic lateral sclerosis, combine expert dysphagia assessment, nutritional optimization, respiratory support, and coordinated oral hygiene to reduce aspiration-related complications and preserve quality of life [69]. Oral nutrition often supports biomechanics of swallowing, sensory aspects of swallowing, and social engagement [70].

In restorative contexts, such as stroke, illness recovery, or temporary deconditioning, tube feeding may serve as a bridge while swallow function improves. In acute stroke, reversible illness, or short-term critical illness when oral intake is clearly inadequate and recovery is expected, temporary enteral nutrition can support hydration, energy and protein delivery, and participation in rehabilitation while swallow function is evaluated and treated. Restorative care (versus palliative care) offers a climate where patients can be challenged and intensive therapeutic strategies can be undertaken, as the patients are expected to recover. Proactive swallow therapy, progressive reintroduction of oral intake when appropriate, and structured oral care are complementary components of recovery. It is unlikely that tube feeding will replace oral feeding long term if rehabilitation of swallowing is consistent with prognosis and patient goals. In contrast, in palliative or life-limiting contexts, nutritional decision-making prioritizes comfort, autonomy, and dignity. Evidence does not demonstrate that tube feeding improves survival or prevents pneumonia in advanced dementia or amid terminal illness. Oral feeding, even when intake is modest, may better align with patient-centered goals, and aspiration risk should not inhibit a patient’s choice to eat by mouth by informed autonomous consent for improved quality of life. Best practices in dysphagia management amid life-limiting illness involve providing guidance on how to deliver food and liquid with caution (e.g., careful hand feeding) and discernment to reduce suffering and lessen risk of high-volume aspiration. Oral care remains essential regardless of the feeding route, as comfort and mucosal integrity are primary concerns.

Eating is not solely a means of nutrient delivery. Eating is a social, cultural, and relational act. Oral feeding supports interpersonal connection, daily structure, and perceived dignity. Removal of oral intake may alter identity and engagement, particularly amid advanced illness. Nutritional decision-making must therefore balance physiological considerations with psychosocial impact [70]. Interdisciplinary collaboration is essential. Dietitians, speech–language pathologists, nurses, physicians, other health care providers, and caregivers each contribute to a patient’s successful tolerance of nutrition. Integrating structured oral care protocols, realistic feeding goals, and shared decision-making supports a care model that is both evidence-based and person-centered. See Table 3 for a visualization of how oral health is linked to nutritional tolerance.

## 4. Discussion

This narrative review synthesizes interdisciplinary evidence to amplify the relationship between oral health and hygiene, pulmonary outcomes, and dysphagia—with specific attention to nutritional decision-making. The findings support a conceptual shift in how aspiration-related pulmonary consequences are understood. Rather than being determined solely by the presence or absence of aspiration events, pulmonary outcomes may be shaped by the interaction between impaired airway protection and the biological characteristics of aspirated material. Oral microbial burden emerges as a central, modifiable contributor to pulmonary vulnerability.

A consistent theme across the literature is that modification of feeding route alone does not eliminate aspiration risk. Aspiration of saliva, secretions, proximally redirected stomach contents (refluxate), or retrograde flow (redirected from esophagus) may occur independent of oral intake. Underlying impairments in airway protection, cough effectiveness, and gastrointestinal function persist regardless of how nutrition is delivered. Past studies investigating oral versus tube feeding amid life-limiting illness (across diagnoses) and dysphagia reveal that tube feeding compared to oral feeding may not reduce pneumonia or mortality and is often associated with increased pulmonary complications. These findings challenge the longstanding belief that bypassing the oropharynx through withdrawal of oral nutrition in favor of enteral nutrition guarantees pulmonary protection. Of note: research reviewed does not indicate that tube feeding is never appropriate. In reversible illness or short-term critical illness when oral intake is clearly inadequate and recovery is expected, temporary and judicious use of enteral nutrition can support hydration, energy and protein delivery, and participation in rehabilitation while swallow function is evaluated and treated.

Oral hygiene is a biologically plausible and clinically actionable pathway for potentially modifying the consequences of aspiration. The oral cavity functions as a dynamic microbial reservoir. Shifts in oral ecology driven by plaque accumulation, reduced salivary flow, and impaired clearance can increase the pathogenicity of aspirated material. Interventions targeting oral microbial burden, including mechanical plaque disruption and the use of adjunctive cleansing agents, have demonstrated reductions in ventilator associated pneumonia in critical-care settings [45,46,48] and are supported by biological and clinical evidence [50,53]. Oral care does not prevent aspiration; however, it can reduce the likelihood that aspiration will result in infectious pulmonary sequelae. Thus, oral hygiene is underscored as a risk-modifying intervention versus simply an activity of daily living.

This narrative review provides a foundation for holistic, nutrition-focused, patient-centered care. Nutritional decision-making when dysphagia is a barrier is often driven by fear of the consequences of aspiration; yet, available evidence reports pulmonary risk to be multidimensional and not mitigated by feeding route alone. Oral health status, salivary adequacy, and the feasibility of maintaining structured oral care should be incorporated into clinical assessment with traditional considerations such as caloric needs, hydration, and disease trajectory. Oral hygiene functions as a primary contributor to nutritional tolerance and feeding sustainability, influencing pulmonary outcomes and also patient comfort and quality of life. The integration of oral care into nutritional decision-making, dysphagia management, and pulmonary protection has interdisciplinary implications. Effective implementation requires coordination between speech–language pathologists, dieticians, nurses, physicians, dentists, endodontists—each contributing to evaluation, protocol development, and ongoing care delivery. This collaborative approach aligns with both restorative and palliative models of care, supporting rehabilitation when recovery is anticipated and prioritizing comfort and dignity when disease is advanced.

A limitation of this work is that it was conducted as a narrative review, emphasizing conceptual analysis and clinical interpretation rather than pooled quantitative effect estimates, so recommendations are best considered guiding principles that may require local adaptation. In addition, available evidence on oral hygiene interventions amid health care complications is uneven, with the strongest data derived from mechanically ventilated and long-term care cohorts; consequently, extrapolation to other clinical settings and diagnostic groups should be made with caution. Key recommendations should not be viewed as definitive practice standards.

**Figure 3:**
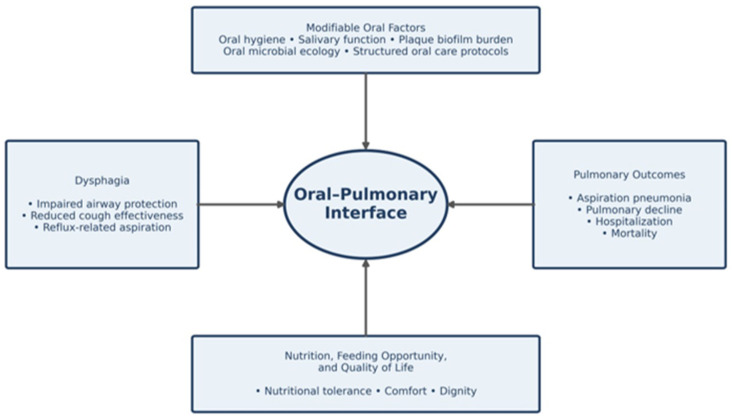
The oral–pulmonary interface amid dysphagia: a conceptual framework.

Literature reviewed encourages a perspective shift related to dysphagia management, adjusting the lens beyond binary feeding decisions toward a layered model, incorporating oral health, microbial ecology, salivary function, and patient goals. Within this model, oral hygiene is positioned as a principal component of pulmonary risk reduction and nutritional care rather than a secondary consideration. See Figure 3.

## 5. Consensus Statements

Based on review of available evidence and clinical experience, the authors propose the following consensus statements. Consensus statements are offered as recommendations to support clinical decision-making and quality improvement and are not approved guidelines.

**Consensus statement 1:**

Withdrawal of oral nutrition in favor of tube feeding may not reliably eliminate risk of aspiration and should not be considered a primary pulmonary-protection strategy in medically-complex or terminally-ill patients. *This conclusion is evidence-based, reflecting cohort studies and systematic reviews comparing outcomes of oral and tube feeding in these populations.

Aspiration pathways persist regardless of feeding route due to ongoing saliva production, secretion management challenges, reflux, and impaired airway protection. Current evidence does not demonstrate consistent reduction in pneumonia incidence or mortality with tube feeding in advanced dementia and life-limiting diagnoses. Nutritional decisions must extend beyond the belief that bypassing oral intake confers pulmonary protection.

**Consensus statement 2:**

Oral hygiene/care is a potentially modifiable contributor to aspiration-associated infectious risk and should be integrated into dysphagia management across care settings. *This recommendation is evidence-based with biologically plausible mechanisms, drawing on clinical trials, systematic reviews, and mechanistic data linking oral biofilm and pulmonary outcomes.

Mechanical plaque disruption, salivary preservation, and targeted antimicrobial oral care modify the pathogenicity of aspirated material. Oral care does not prevent aspiration. It mitigates the consequences. Structured oral hygiene represents an evidence-informed intervention in acute care, long-term care, rehabilitation, and palliative environments.

**Consensus statement 3:**

Nutritional decision-making in dysphagia must be patient-centered and integrative—analyzing and weighing pulmonary risk, oral health and viability, functional prognosis, and quality-of-life. *This statement represents expert consensus grounded in the available evidence, rather than a prescriptive practice guideline.

Oral feeding supports neuromuscular activation, sensory engagement, social connection, and dignity. In restorative contexts, rehabilitation should be prioritized. Amid life-limiting illness, comfort and autonomy may supersede feared consequences of aspiration. Oral hygiene remains essential regardless of feeding route.

## 6. Future Directions and Research

Future research should prioritize prospective and controlled investigations examining the impact of structured oral hygiene interventions across diverse dysphagia populations, including: (a) patients who are not mechanically ventilated; (b) residents in inpatient rehabilitation, skilled nursing, or long-term care facilities, and (c) individuals receiving palliative care. Future research should also continue to address the effects of oral cleansing best practices in tertiary care settings. Moreover, it would be fruitful to investigate the impact of routinized oral care on outcomes beyond pneumonia and pulmonary health, such as hospitalization, nutritional status, feeding tolerance, and quality of life. Further work is needed to clarify optimal oral care protocols (e.g., frequency, intensity, and comparative effectiveness of specific cleansing agents). Direct comparisons between commonly used agents, such as chlorhexidine and hydrogen peroxide and combination protocols, may help to refine evidence-based approaches that can be standardized and implemented across care settings. Investigation into dose–response relationships and long-term safety profiles will be particularly important. Multi-center studies may help identify which protocol components are most effective and generalizable across different care environments.

The role of saliva in moderating pulmonary risk remains an important area for future investigation. Studies evaluating interventions that maintain or enhance salivary flow through behavioral, pharmacologic, or supportive care strategies may better elucidate the relationship between oral physiology and aspiration-related pulmonary decline. Integration of oral health assessment into established nutrition and dysphagia care pathways also warrants investigation. Incorporating standardized oral health metrics into nutritional risk screening tools and dysphagia evaluation protocols may improve identification of patients with elevated pulmonary risk and facilitate earlier, targeted oral care. Implementation research is also needed to determine how oral care pathways can be integrated into interdisciplinary dysphagia intervention. Finally, interdisciplinary models of care that formally incorporate dental expertise into hospital and long-term care settings should be reviewed and considered for feasibility, scalability, and clinical impact. Understanding how best to operationalize oral care within existing health care infrastructures will be critical to translating evidence into routine practice.

## 7. Conclusions

The relationship between dysphagia and pulmonary health extends beyond the mechanics of swallowing. Pneumonia risk emerges from a complex interaction between impaired airway protection, oral microbial ecology, salivary function, systemic disease burden, and feeding practices. The microbial characteristics of aspirated material critically influence pulmonary outcomes.

Evidence reviewed in this manuscript supports weighing use of tube feeding in the context of nutritional-decision making, as its use may not reliably prevent pneumonia or improve survival in advanced illness. Aspiration of saliva and refluxate may continue to occur after patients or caregivers have been advised that cessation of oral feeding with risk of aspiration replaced by tube feeding is safer and protective of the lungs. Early respiratory morbidity following tube placement can occur and is well documented amid patients with life-limiting conditions. Rather, routine oral hygiene with evidence-based agents and mechanical disruption (brushing) is an emerging ally to the reduction in pneumonia risk via decreasing the pathogenic load of aspirated secretions.

Prioritizing oral care regardless of feeding route elevates pulmonary health as an important consideration. Key components are: (a) consistent oral care, (b) maintaining salivary function, (c) collaboration among health care providers, and (d) uplifting decisions that reflect individual patient priorities. Priorities apply across restorative and palliative care settings. Embedding structured oral care into interdisciplinary practice offers a pragmatic and evidence-aligned pathway to improve respiratory outcomes, preserve feeding opportunity, and enhance quality of life for individuals with dysphagia.

## Figures and Tables

**Table 1:** Oral hygiene agents and mechanisms relevant to pulmonary risk modification.

Agent/Modality	Mechanism of Action	Evidence Context	Reported Findings (As Described in Manuscript)
Chlorhexidine (CHX; 0.01–0.2%)	Broad antimicrobial activity; substantivity to oral tissues; biofilm disruption	ICU and mechanically ventilated populations	Meta-analysis ranked brushing + 0.12% CHX most effective
Hydrogen peroxide (1–1.5%; temporarily 3%)	Oxidizing agent; disrupts bacterial membranes; reduces anaerobic organisms; loosens debris	ICU randomized trials; dental literature	RCT showed reduced VAP versus saline; review supports temporary 3% use; concentrations > 1% associated with plaque/gingivitis reduction
Sodium bicarbonate (NaHCO_3_)	Alkalinizes oral cavity; thins secretions; facilitates mechanical debris removal	Multi-component ICU and neonatal oral care bundles	NaHCO_3_ + CHX associated with lower VAP incidence versus CHX alone; used in neonatal bundled protocol
Tooth brushing (soft bristle)	Mechanical plaque biofilm disruption; reduces microbial density	ICU oral care protocols; meta-analysis	Brushing combined with CHX ranked highest for VAP prevention; brushing alone also beneficial
Oral moisturization/salivary support	Maintains mucosal hydration; supports antimicrobial salivary function	Salivary physiology literature	Hyposalivation associated with increased bacterial density and biofilm accumulation

**Table 2:** Structured oral care protocol components.

Protocol Component	Recommended Frequency	Targeted Physiologic Effect	Applicable Feeding Route
Mechanical plaque disruption (tooth brushing; include gingival margin and tongue dorsum)	Twice daily/every 12 h	Biofilm disruption; reduction in microbial burden	Oral and tube feeding
Oral cleansing with antimicrobial or oxidizing agent (e.g., CHX 0.01–0.2%; hydrogen peroxide 1–1.5%)	Every 2–4 h (at minimum every 12 h)	Reduction in pathogenic load in secretions; clearance of debris	Oral and tube feeding
Post-meal oral cavity cleansing	After meals	Removal of food residue; reduction in aspirated bacterial load	Oral feeding
Salivary preservation/oral moisturization	Ongoing routine care	Maintenance of mucosal integrity; modulation of oral microbiome	Oral and tube feeding
Denture removal and cleaning; overnight storage outside mouth	Daily; remove overnight	Reduction in denture-associated biofilm colonization	Oral and tube feeding
Dental assessment/oral disease triage (cavitated caries, periodontitis, retained roots, sharp edges, suspected odontogenic infection)	On admission; repeat as indicated (pain, feeding refusal, oral lesions)	Identifies and addresses oral pain and biofilm-retentive foci that impair mastication, oral intake tolerance, and adherence to oral care	Oral and tube feeding

Typical concentrations, frequencies of administration, and protocol durations for commonly used oral cleansing agents (e.g., chlorhexidine, hydrogen peroxide, sodium bicarbonate) are summarized based on available clinical studies and practice protocols.

**Table 3:** Mechanistic pathways linking oral hygiene to nutritional tolerance in dysphagia.

Oral Health Variable	Physiologic Consequence	Impact on Swallow/Secretion Management	Implication for Nutritional Tolerance
Adequate salivary flow	Buffers pH; dilutes microbes; supports antimicrobial defense	Improves bolus lubrication and cohesion	Supports effective and functional oral intake and oral feeding tolerance
Hyposalivation/xerostomia	Increased bacterial density; accelerated plaque accumulation; altered microbiome	Impaired bolus formation; increased secretion viscosity	Reduced comfort and tolerance for oral feeding; increased pulmonary vulnerability
Mechanical plaque disruption	Reduces biofilm burden	Lowers pathogenic load of secretions (or food and drink) that may be aspirated	Decreases biological risk of aspiration during oral feeding
After-meal cleansing	Clears food residue; reduces bacterial colonization	Reduces retained debris in oral cavity	Supports repeated oral intake opportunities through preserving pulmonary stability
Denture hygiene	Reduces denture-associated biofilm colonization	Improves oral comfort; reduces colonized surfaces	Facilitates oral feeding participation and inhibits pulmonary ingestion of oral pathogens
Continued oral feeding (versus withdrawal)	Maintains neuromuscular activation; preserves salivary stimulation	Supports swallow frequency, neuroplasticity, and secretion clearance	May preserve feeding tolerance, reduce deconditioning, and limit harm from tube-feeding associated pneumonia or mortality
Oral pain/active dental disease (caries, retained roots, odontogenic infection	Pain/inflammation; biofilm-retentive niches; reduced tolerance/compliance with oral care	Impaired mastication and oral-phase control; texture avoidance; increased oral residue/poor clearance	Reduced oral intake/diet variety; increased refusal/nonadherence; delayed functional oral feeding unless dental issues addressed

## Data Availability

No new data were created or analyzed in this study. Data sharing is not applicable to this article.
